# Semantic Cues Modulate Children’s and Adults’ Processing of Audio-Visual Face Mask Speech

**DOI:** 10.3389/fpsyg.2022.879156

**Published:** 2022-07-19

**Authors:** Julia Schwarz, Katrina Kechun Li, Jasper Hong Sim, Yixin Zhang, Elizabeth Buchanan-Worster, Brechtje Post, Jenny Louise Gibson, Kirsty McDougall

**Affiliations:** ^1^Faculty of Modern and Medieval Languages and Linguistics, University of Cambridge, Cambridge, United Kingdom; ^2^Medical Research Council Cognition and Brain Sciences Unit, University of Cambridge, Cambridge, United Kingdom; ^3^Faculty of Education, University of Cambridge, Cambridge, United Kingdom

**Keywords:** speech processing, face masks, cued shadowing, audio-visual integration, semantic prediction, language development, internet-based data collection, bottom-up vs. top-down

## Abstract

During the COVID-19 pandemic, questions have been raised about the impact of face masks on communication in classroom settings. However, it is unclear to what extent visual obstruction of the speaker’s mouth or changes to the acoustic signal lead to speech processing difficulties, and whether these effects can be mitigated by semantic predictability, i.e., the availability of contextual information. The present study investigated the acoustic and visual effects of face masks on speech intelligibility and processing speed under varying semantic predictability. Twenty-six children (aged 8-12) and twenty-six adults performed an internet-based cued shadowing task, in which they had to repeat aloud the last word of sentences presented in audio-visual format. The results showed that children and adults made more mistakes and responded more slowly when listening to face mask speech compared to speech produced without a face mask. Adults were only significantly affected by face mask speech when both the acoustic and the visual signal were degraded. While acoustic mask effects were similar for children, removal of visual speech cues through the face mask affected children to a lesser degree. However, high semantic predictability reduced audio-visual mask effects, leading to full compensation of the acoustically degraded mask speech in the adult group. Even though children did not fully compensate for face mask speech with high semantic predictability, overall, they still profited from semantic cues in all conditions. Therefore, in classroom settings, strategies that increase contextual information such as building on students’ prior knowledge, using keywords, and providing visual aids, are likely to help overcome any adverse face mask effects.

## Introduction

Listening to speech produced through face masks can sometimes reduce intelligibility, especially when listening to masked speech in noise (Bottalico et al., 2020; Brown et al., 2021). These findings have caused some uncertainty around the impact face masks have on communication and have led to debates on the use of face masks in educational settings in particular (UK Government, Department for Education, 2022). However, the extent of processing difficulties and whether they are predominantly caused by changes to the acoustic signal or by the visual obstruction of the speaker’s mouth are unclear. In addition, it has not been determined whether any such speech processing difficulties are present in all semantic contexts or only when the conversation content is unpredictable (*cf*. Randazzo et al., 2021). Face mask research has so far predominantly concentrated on adults’ perception of masked speech, not children’s, despite evidence that language processing mechanisms change across the lifespan (Wightman et al., 2006; Mahler and Chenery, 2019). The present study addresses these gaps with a novel, internet-based cued shadowing task developed to capture precise response times to masked speech and compares the interaction between acoustic, visual, and semantic cues when children and adults process language produced through face masks.

A growing body of research has tried to capture the subtle acoustic changes caused by different types of face coverings. The most commonly found effects are reduced intensity of the speech signal and changes to higher frequencies, the exact threshold depending on the setup, and type of face covering used. Llamas et al. (2008) report that out of a niqāb, a balaclava, and a surgical mask, only the surgical mask significantly attenuated frequencies between 2.5 and 12.5 kHz as well as between 14 and 24 kHz. Newer studies include a wider variety of commonly used face masks in the context of COVID-19 and report varying transmission loss above different thresholds depending on mask type and measurement (e.g., above 1 kHz, Nguyen et al., 2021; above 2 kHz, Pörschmann et al., 2020; above 3 and 5 kHz, Magee et al., 2020), with especially high acoustic degradation observed for transparent masks (Corey et al., 2020).

These acoustic changes are likely caused by the dampening effect of face masks and by the restriction of the articulators. Despite these effects, the acoustic changes described seem to affect the accuracy of speech recognition (“intelligibility”) only minimally. Llamas et al. (2008) report that few misperceptions (less than 2%) occurred when participants were asked to write down the target words of carrier sentences. More misperceptions occurred when participants listened to a female voice (*ca*. 70%, compared to a male voice). Overall, only a few mistakes were made, of which the most common included confusing stops with fricatives (e.g., /t/~/θ/), the place of articulation (e.g., /k/~/p/) and voicing of stops (e.g., /p/~/b/), and the place of articulation of fricatives (especially /f/~/θ/) and of nasals (especially /n/~/ŋ/; Llamas et al., 2008). However, recent research found no significant effect of face masks on single word or sentence intelligibility in quiet conditions (Magee et al., 2020; Brown et al., 2021).

Given the absence of strong effects in optimal listening conditions, several studies have examined adults’ masked speech perception in noise (Bottalico et al., 2020; Brown et al., 2021; Randazzo et al., 2021; Smiljanic et al., 2021; Truong and Weber, 2021). These studies all tested speech intelligibility in different types of moderate to high levels of noise by asking participants to type what an adult speaker had said, sometimes paired with rating the listening effort. All studies found that masks reduced adults’ accuracy in identifying words and sentences under noisy conditions, with some variation depending on mask type and noise level (for details see, e.g., Bottalico et al., 2020; Brown et al., 2021; Randazzo et al., 2021). In addition to intelligibility effects, Bottalico et al. (2020) and Brown et al. (2021) found that all mask types led to higher ratings of listening effort, and Truong and Weber (2021) showed that fabric masks affected recall performance under 12 dB SNR, with participants recalling fewer words when speakers were wearing a mask (55% compared to 59% when no mask was worn; see also Truong and Weber, 2021, for reduced memory performance of masked speech in quiet conditions).

The acoustic effects of face masks on children’s speech processing, to the authors’ knowledge, have not yet been tested. Past research on children’s processing of speech indicates that they may be affected more markedly by degradation of the acoustic signal than adults (Talarico et al., 2007; Klatte et al., 2010, 2013), possibly due to the fact that the auditory cortex continues to develop until adolescence (Paus et al., 1999). As such, the acoustic changes caused by face coverings might affect children more strongly than adults, especially in classroom contexts, where the presence of noise might further add to speech comprehension difficulties. Furthermore, only a few studies have tested masked speech processing with more naturalistic paradigms than typing and subjective ratings. Using the Bamford–Kowal–Bench task, where participants repeat the target words of spoken sentences (more widely known as shadowing), Hampton et al. (2020) found a significant reduction of speech discrimination performance under simulated hospital noise of 70 dB. However, they tested only five participants, and in line with other studies, the authors collected accuracy data, but no reaction times. Given the lack of evidence that masked speech leads to reduced intelligibility in quiet conditions despite anecdotally reported difficulties in understanding face mask speech, mild processing costs in conditions with low background noise may be captured more effectively by measuring reaction times.

The majority of face masks create a visual barrier to the lower part of a speaker’s face, preventing access to visual cues from a speaker’s lips. However, visual cues from the speaker’s eyes are still available, and listeners (both infants and adults) have been found to pay more attention to a speaker’s eyes than their mouth in quiet situations (Vatikiotis-Bateson et al., 1998; Smith et al., 2013). Despite this, Jordan and Thomas (2011) found that only occluding the lower half of the face significantly affected auditory response accuracy when identifying sounds involving bilabial and labiodental articulations, whereas covering the upper half of the face did not. Moreover, visual cues from the mouth become more important when the audio-signal is affected, e.g., by noise (Sumby and Pollack, 1954; Lansing and McConkie, 2003), leading adults to shift their attention from the eyes to the mouth (Vatikiotis-Bateson et al., 1998). Yi et al. (2013), for example, show that fixations close to the speaker’s mouth were more frequent under a low speech-signal-to-noise ratio (SNR) than a high speech-SNR. This suggests that peripheral vision of a speaker’s mouth is insufficient under challenging conditions.

Besides noise, many other factors can make speech processing more demanding. As Reisberg et al. (1987) argue, visual cues are integral to all audio-visual speech processing (also *cf*. Arnold and Hill, 2001), but given near-perfect performance under auditory processing alone, visual benefits only become apparent as cognitive demand increases, for example when adults shadow complex philosophical passages (Experiment 4) or when novice L2 learners shadow speech in the foreign language (Experiments 1 and 2). In line with these findings, Barenholtz et al. (2016) showed that adults attend more to the mouth in response to an unfamiliar language compared to a familiar one, and Birulés et al. (2020) found that even highly competent L2 speakers look more at the speaker’s mouth when presented with nonnative speech. Furthermore, Yi et al. (2013) presented two talker faces with speech from one of the speakers. When participants’ fixations were close to the speaker’s mouth (within 2.5 degrees of the center of the mouth) in the more demanding two-talker image condition, speech intelligibility was similar to fixations within 10 degrees from the mouth of a single talker image. Taken together, these studies suggest that under cognitively demanding processing conditions adults rely more on audio-visual speech cues and as a result shift their attention from the eyes to the mouth of the speaker to aid speech processing.

Moreover, the integration of auditory and visual speech processing is not simply the sum of the combined cues, but instead exhibits a complex, nonlinear relationship. Although the integration of visual and auditory information is greatest in moderately noisy conditions (Ross et al., 2007; Ma et al., 2009), indicating multisensory enhancement for imperfect acoustic signals (principle of inverse effectiveness, Stein and Meredith, 1993; van de Rijt et al., 2019), visual processing deteriorates in excessive noise (van de Rijt et al., 2019). Face masks can be considered a type of mild acoustic noise that in principle could induce a shift of attention from the eyes to the mouth; however, since most masks are opaque, they also prevent access to lip-reading, potentially adding to the difficulty of perceiving face mask speech. It is therefore unclear what effects face masks have on each modality individually as well as on the combined audio-visual process.

Audio-visual speech perception starts to develop early from infancy (Lewkowicz, 2010; Lewkowicz et al., 2015). Infants in general pay more attention to the eyes when attending to a speaker, but attention to the mouth starts to increase from six months of age, around the time they start to babble and learn to speak (Teinonen et al., 2008; Lewkowicz and Hansen-Tift, 2012; Tenenbaum et al., 2013). Toddlers who are able to shift their gaze from the speaker’s eyes to the mouth under noisy conditions show higher word recognition (Król, 2018), and 5–11-year-old children, both with and without language learning impairments, benefit from visual cues from the talking face, especially when listening to speech in noise as shown by Knowland et al. (2016), for example. However, Wightman et al. (2006) tested speech perception in children and adults while simultaneously displaying a time-synchronized distractor message and reported that the addition of a video of the speaker improved older children’s (aged 12–16) and adults’ performance more than younger children’s (aged 6–11). In line with this, Maidment et al. (2015) found that children younger than 6 years of age do not utilize visual speech cues for speech processing to the same degree as adults when the acoustic signal is degraded. This indicates that children benefit from visual cues early on, but continue to develop an integrated audio-visual processing system until adolescence.

Face masks typically cover only the lower half of the face, and therefore, listeners are still able to pick up cues from the eyes, upper half of the face, and slight movements of the mask. Llamas et al. (2008) found that fewer mistakes were made in their single-word perception test when the speaker’s video was presented together with the audio (0.6% mistakes out of all responses in the audio-visual condition compared to 0.8% in the audio-only condition), indicating that missing visual cues could play a vital role in face mask-related speech misperceptions. Zhang et al. (2021), however, found little to suggest that seeing a speaker’s lips significantly improved speech perception. Specifically, their study tested how masks affect perception of words featuring prominent visual lip-rounding cues, comparing minimal pairs such as “see/she,” where the latter was produced with a visually more rounded shape of the lips than the former. Stimuli were visually manipulated by displaying the speaker with/without mask and acoustically by presenting the audio signal produced with/without a mask. Native and nonnative English speakers then had to choose which word they heard. Although nonnative listeners made more mistakes and had longer response latencies than native speakers, this effect was not driven by the visual or acoustic mask manipulations, suggesting that visual cues in fact play only a minor role for accurate and efficient perception of sounds involving lip-rounding. It remains unclear to what extent visual cues could be advantageous in processing masked speech when words are presented in sentence contexts, as is the case in natural listening conditions.

In addition to acoustic and visual information, utilizing prior knowledge of phonological, lexical, syntactic, and semantic constraints is vital to processing connected speech efficiently (Kuperberg and Jaeger, 2016). For instance, high semantic predictability of sentence-final words (also known as Cloze Probability, *cf*. Liu et al., 1997) aids rapid speech processing as it allows cortical circuits to probabilistically predict and pre-activate upcoming content based on semantically related information presented earlier in the sentence (Kalikow et al., 1977). Decades of research have demonstrated the importance of semantic predictions in speech processing (Kamide et al., 2003; Klimovich-Gray et al., 2019). Aydelott et al. (2006) found that acoustic degradation modulated neurophysiological responses to semantic congruence in their study. When the unaltered speech signal was presented, the N400 component amplitude was significantly smaller for semantically congruent than incongruent words. Presenting stimuli with a low-pass sound filter, however, reduced the difference between conditions (i.e., indicating reduced availability of semantic information) despite highly accurate responses. In contrast to this finding, a number of studies have shown that predictability aids processing in adverse listening conditions (e.g., McGettigan et al., 2014; Sohoglu and Davis, 2016), and as such one might expect greater reliance on and use of semantic information when less detailed acoustic information is available. This raises questions about the use of semantic information when processing masked speech, since face mask speech presents one type of challenging listening condition due to the degraded acoustic signal and reduction of visual cues. However, to the best of the authors’ knowledge, no study to date has investigated to what extent semantic predictability modulates face mask effects.

For children, the extent to which semantic context can be beneficial when they are faced with degraded visual and auditory mask input also remains unclear. This question is not only practically relevant given that face masks affect visual and acoustic speech cues in an already noisy classroom environment (see Picard and Bradley, 2001), but also theoretically interesting since the degree to which semantic cues facilitate comprehension could depend on the developmental language stage. Research indicates that children develop their linguistic skills throughout childhood, including semantic processing mechanisms (Hahne et al., 2004; Atchley et al., 2006; Lewis et al., 2010). This could affect the way they rely on and integrate semantic cues with acoustic and visual input relative to adults. Despite indications that children are able to use semantic cues to some extent by the age of two years (Friedrich and Friederici, 2004), there is currently no consensus as to whether the ability to use contextual information in adverse listening conditions differs between children and adults (e.g., Elliott et al., 1979; Fallon et al., 2002). Growing evidence indicates that contextual processing becomes more efficient with increasing age (Atchley et al., 2006). However, it is unclear how much of this is due to developing processing mechanisms (including attentional and inhibitory processes) or lack of experience (Mahler and Chenery, 2019). Little is known about the effect of face masks on semantic processing, and whether children are able to use semantic predictions to compensate for the degraded signal to the same extent as adults when listening to a teacher wearing a face mask. As discussed earlier, attention is shifted from a talker’s eyes to their mouth as speech processing becomes more demanding (e.g., L2 processing, Birulés et al., 2020). Optimized audio-visual integration is therefore likely to be more important in unpredictable semantic contexts when top-down compensation is unavailable as this presents a more cognitively demanding situation.

The current study tested (1) the (inhibiting) effects of acoustic and visual face masks, (2) how these effects are modulated by semantic predictability, and (3) how children integrate these linguistic cues compared to adults. Given the absence of intelligibility effects under optimal listening conditions (i.e., in the absence of background noise) in previous studies (e.g., Brown et al., 2021), significant effects of face masks were predicted for response latencies (reaction time), but not necessarily for response accuracy. For the present study, an internet-based version of the cued shadowing task (also known as “auditory word repetition,” Bates and Liu, 1996; Liu et al., 1997) was developed. This task was used to capture millisecond-accurate response latencies (vocal response times) to audio-visual recordings of face mask speech and unmasked speech in order to shed light on the time-course of masked speech processing. Cued shadowing requires participants to repeat the last word (the target) of sentences, which were presented in synchronized audio-visual format in the current study. A priming effect can be measured when sentence manipulation leads to faster repetition of the target word, reflecting faster processing. Shadowing does not require literacy or metalinguistic processes and as such serves as a naturalistic measure of listening effort, i.e., of the resources required by a listener to meet the cognitive demands of processing speech accurately and efficiently (*cf*. Houben et al., 2013; Peelle, 2018). As such, the method is well-suited to both children and adults (Mahler and Chenery, 2019). Collecting high-precision naming latencies has recently been successfully adapted and validated for internet-based data collection using picture naming (Fairs and Strijkers, 2021; Vogt et al., 2021). Building on this success, the present study collected response latencies through cued shadowing *via* the internet for the first time.

In the current study, stimuli were manipulated with an Acoustic Mask, a Visual Mask, and varying semantic predictability. The third variable, semantic predictability, was captured by manipulating the Cloze Probability of the target word, i.e., the “probability that the target is predictable given a sentence context” (Liu et al., 1997, p. 165). For example, the target word “cake” has high Cloze Probability in the sentence “*For your birthday I baked this cake,*” but low probability in the sentence “*Tom wants to know about this cake*” (Kalikow et al., 1977). Through predictive speech processing (i.e., anticipating upcoming linguistic input through activating prior knowledge), listeners on average respond faster to stimuli with high Cloze Probability (Liu et al., 1997). Therefore, in the present study, it was hypothesized that adult listeners would utilize semantic predictions to compensate for any adverse Acoustic and Visual Mask effects, leading to smaller response latency differences between masked and unmasked conditions when the target word can be predicted from the carrier sentence. Since children have less experience in using semantic cues for predictive speech processing as well as less fine-tuned cognitive control mechanisms (at least below age 13; Atchley et al., 2006), it was hypothesized that they might integrate acoustic, visual, and semantic cues differently from adults. However, additional visual cues from the speaker’s mouth were expected to reduce processing difficulties significantly for both children and adults. Finally, it was expected that processing masked speech would become less effortful with practice, which would be reflected in faster responses to masked speech toward the end of the experiment.

In sum, the present study tested the following three main predictions:

*P*1: Significant effects of face masks are present for response latencies (reaction time), but not for response accuracy.

*P*2: Semantic cues, visual cues, and acoustic cues all help reduce the processing difficulties significantly for both children and adults.

*P*3: Adults are more proficient in utilizing and integrating semantic, visual, and acoustic cues than children.

## Materials and Methods

### Participants

Participants were recruited through primary schools in Cambridgeshire and Greater London as well as through social media between May and September 2021. After screening for participants who did not show an honest commitment to the task (e.g., no response in all or most trials, attempt to complete the study more than once, loud background noise or conversations unrelated to the experiment throughout, *cf*. Fairs and Strijkers, 2021), 68 adults and 63 children living in the United Kingdom had completed the experiment. Three adults and three children were excluded because they did not meet the specified recruitment criteria, and 25 adults and 33 children were excluded because of insufficient technical equipment or suboptimal recording quality. The remaining 26 children (aged 8–12, *M* = 10.9, *f* = 15, *m* = 11) were matched in number with 26 randomly selected adults (aged 20–60, *M* = 30.8, *f* = 17, *m* = 9). All participants had acquired English before the age of seven and reported having no hearing, writing or language difficulties. Twenty-three adults and twenty-one children were monolingual English speakers, and three adults and five children were bilingual (English and another language).

A short questionnaire collected information about participants’ experience with face masks. The majority of adult participants self-reported that they “sometimes” or “frequently” have had problems with understanding people who wear a face mask (85%), while 15% reported no problems. In contrast to this, only 42% of parents reported that their child had sometimes encountered problems with understanding people with a face mask, while 42% answered that they had not encountered any such problems (15% answered “I do not know”). Adult participants also judged masked speech as more difficult and more tiring than parents did for their children: On a scale of 1 (very easy) to 5 (very difficult), adults rated the difficulty of understanding masked speech higher on average (*M* = 2.7, SD = 0.69, Mdn = 3.0), than parents on behalf of their children (*M* = 2.3, SD = 0.72, Mdn = 2.0). On a scale of 1 (not tiring) to 5 (very tiring), adults also rated listening to masked speech as more tiring (*M* = 2.4, SD = 1.10, Mdn = 2.5) than parents on behalf of their children (*M* = 2.0, SD = 1.00, Mdn = 2.0).

Ethical approval was obtained from the University of Cambridge Faculty of Modern and Medieval Languages and Linguistics Research Ethics Committee. Informed consent was obtained from all adult participants and children’s parents. Adult participants and families of child participants received, respectively, £5 and £10 supermarket vouchers for their participation.

### Materials

A total of 240 sentence stimuli were selected from Kalikow et al. (1977). Each of the 120 target words was embedded at the end of a high and a low predictability carrier sentence with five to eight words and six to eight syllables, thereby manipulating the Cloze Probability of the target words (e.g., “*For your birthday I baked this cake*” vs. “*Tom wants to know about this cake*”). Stimuli were adapted where necessary to ensure that sentences were compatible with British English and that the target words were always preceded by the same phonological environment (full list of stimuli in Supplementary Material 1). All target words were neither highly frequent nor infrequent (5 to 150 pmw, Thorndike and Lorge, 1952) and had a mean age of acquisition of eight years or younger (Kuperman et al., 2012). In addition to Cloze Probability, stimuli were manipulated by an Acoustic Mask (speech produced through mask vs. no mask) and a Visual Mask (speaker wears a mask in the video vs. no mask). All three factors (Acoustic, Visual, and Cloze Probability) were fully crossed, leading to a 2 × 2 × 2 within-item design (Table 1). In practice, this meant that participants were presented with low and high Cloze Probability sentences:

1. where there was no mask present for either sound or video (–Acoustic Mask, –Visual Mask),
2. where the sound was produced with a mask, but the video displayed the speaker without a mask (+Acoustic Mask, –Visual Mask),
3. where the sound was produced without a mask, but the video displayed the speaker wearing a mask (–Acoustic Mask, +Visual Mask), and
4. where a mask was present for both sound and video (+Acoustic Mask, +Visual Mask).

**Table 1:** Experiment conditions.

	–Visual mask	+Visual mask
**–Acoustic mask**	(1) High vs. low probability^*^	(3) High vs. low probability
**+Acoustic mask**	(2) High vs. low probability	(4) High vs. low probability

* Examples: For your birthday I baked this cake. (High)/Tom wants to know about this cake (Low).

For the simultaneous audio-video recordings, a female native English speaker with Standard Southern British English accent, who was also a trained phonetician, spoke the stimuli with and without a cloth mask. To ensure a similar speaking rate and intonation across all conditions, the speaker imitated her own production of the stimuli, which had previously been recorded and were then played back to her. This was essential for the audio-to-video synchronization across conditions, as well as for avoiding uncontrolled phonetic cues that might affect perception.

The mask was hand-made with two layers of 100% cotton, a type of mask commonly used in the United Kingdom. According to previous studies which have examined acoustic effects of comparable two-layered cotton masks, cloth masks, like other kinds of face masks, mainly reduce energy at high frequencies, whereas frequencies below 1 kHz are largely unaffected. Magee et al. (2020), for example, examined the acoustic changes of a comparable two-layered cotton mask (among other mask types), and found that power (dB/Hz^2^) of the speech signal was significantly lower between 5 and 10 kHz compared to no mask. Other measurements such as *f*0, intensity, as well as voice quality measures such as harmonic-to-noise ratio and cepstral peak prominence were not significantly affected. Although the amount of attenuation varies depending on the measurement setting, it has generally been shown that the acoustic performance of cloth masks is better than the performance of N95 masks, but worse than surgical masks (Corey et al., 2020; Brown et al., 2021; Toscano and Toscano, 2021).

**Figure 1:**
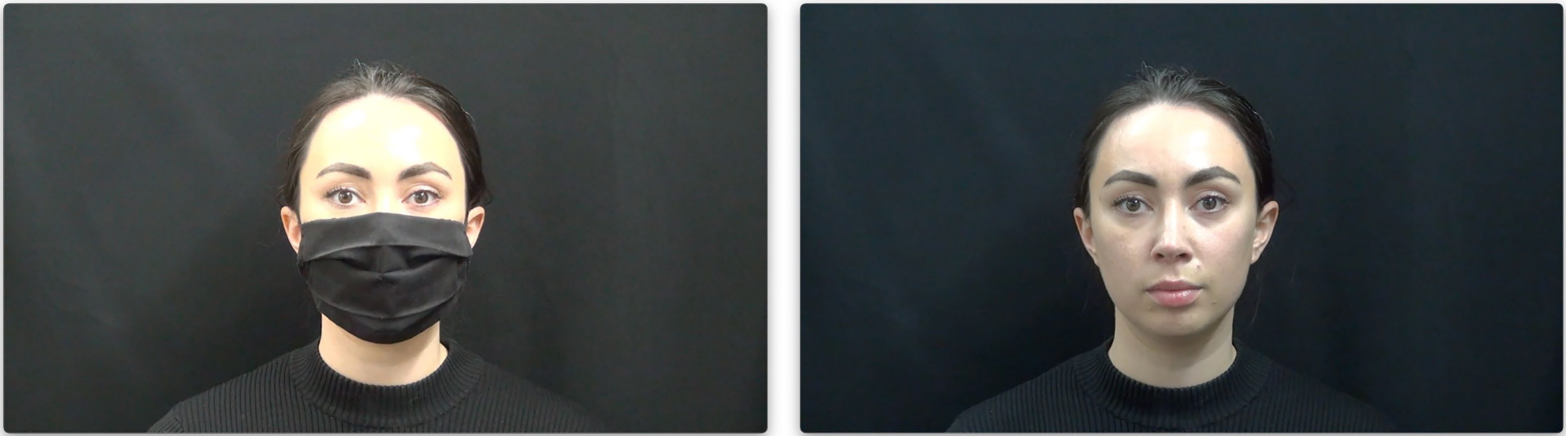
The speaker with and without a face mask in neutral position.

Audio and video recordings were made simultaneously in a sound-attenuated recording booth with a black backdrop as the video background. The audio stimuli were recorded with a Zoom H4n recorder at a sampling rate of 44,100 Hz at 16 bits, and the visual stimuli were recorded with a SONY Handycam HDR-PJ580 20.4 Megapixels, with 1,920-by-1,080 resolution at 50 frames per second. The audio and video files were synchronized and sliced in Final Cut Pro (version 10.5.2). Each video was cross-dubbed with the masked and unmasked sound recordings while carefully maintaining synchrony between sound and image. Silence before and after each stimulus ensured the speaker’s mouth started from and returned to a neutral position (*cf*. Figure 1).

**Figure 2:**
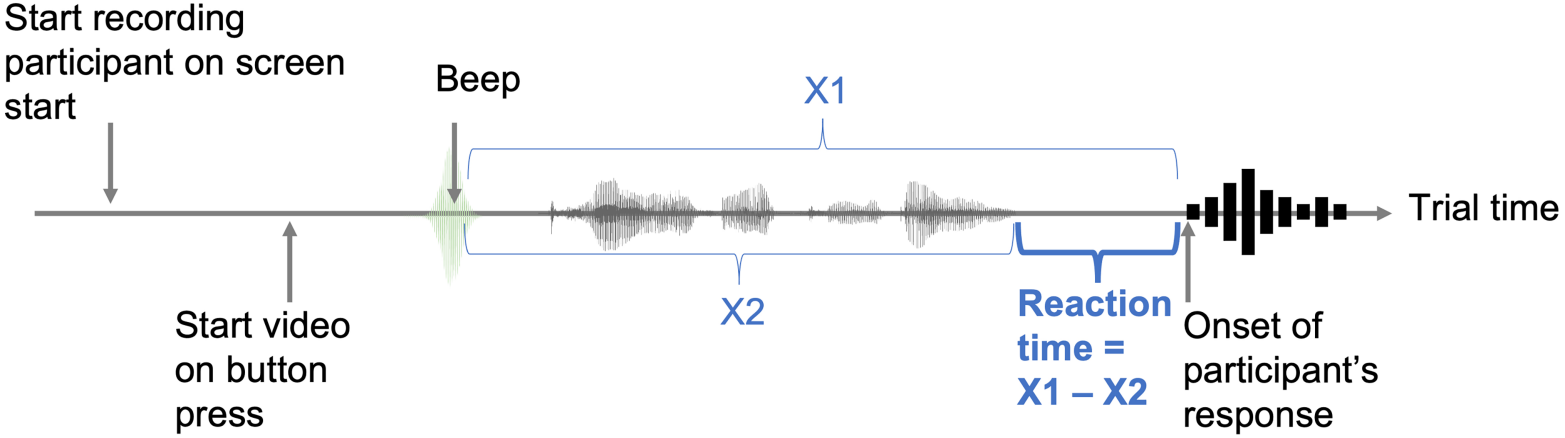
Trial design for capturing reaction times (RT). X1: Duration of trial recording from the beep to the response onset. X2: Duration of stimulus from the beep to the end of the presented sentence.

Finally, a single-peak acoustic signal (henceforth referred to as “beep”) generated in *Praat* (Boersma and Weenink, 2022) was inserted at the beginning of each stimulus sentence to serve as an anchor for measuring reaction times. Crucially to this design, the audio recordings collected in the experiment started from the beginning of each trial, thereby capturing both the sound signal (marking the beginning of the trial) and the participant’s vocal response in the same recording. To obtain the response time for each trial, the duration of the sentence stimuli was later subtracted from the duration between the peak of the beep and the onset of the participant’s response (Figure 2).

### Procedure

The study was prepared with Gorilla experiment builder (Anwyl-Irvine et al., 2020). The shadowing experiment was preceded by the study information and ethical consent form, a questionnaire collecting demographic data and participants’ experience of masked speech communication, and calibrations for sound (speaker test) and voice recording (microphone test). Parents completed the questionnaires and consent forms for their children, but children performed the calibration and shadowing experiment themselves.

Children and adults were randomly assigned to one of eight counter-balanced, pseudo-randomized versions of the shadowing experiment to ensure equal distribution of the conditions and that participants saw each of the 120 target words only once. Before the start of the experiment, participants were asked to sit in front of a laptop or desktop screen that was placed on a desk or table. They were instructed to use speakers so that the output of the audio stimuli, especially the beep marking the beginning of each trial, would be captured in the recording. Participants then received written instructions to repeat the last word of sentences as quickly and as accurately as possible, followed by an animated demonstration of the task. In addition, participants were instructed to say “I do not know” if they did not know the correct answer. The children’s version of the experiment introduced a parrot named Polly and asked the children to “teach” Polly new words.

The experiment started with 12 practice items and a reminder of the instructions. 120 trials were equally distributed across four blocks. After each block, the participant was shown their progress through the total number of trials and given the opportunity to take a break. Each trial began with a 250 ms fixation cross, followed by the audio-visual display of a sentence, which was manually started by the participant upon clicking on the video. Participants then gave their vocal response before clicking the “Next” button. A progress bar and the correct answer were displayed on the screen for 1.5 s (by Polly the parrot) before automatically continuing to the next trial.

After finishing the shadowing task, the adult participants and the children’s parents were asked to rate the difficulty of the experiment on a five-point scale from 1, “very easy,” to 5, “very difficult” (adults: *M* = 1.7, SD = 0.87, Mdn = 2.0; children: *M* = 1.8, SD = 0.61, Mdn = 2.0), and their concentration during the study on a five-point scale from 1, “very concentrated,” to 5, “very distracted” (adults: *M* = 1.7, SD = 0.74, Mdn = 2.0; children: *M* = 1.7, SD = 0.85, Mdn = 1.0). The shadowing experiment took each participant *ca*. 20–25 min, and the whole study *ca*. 30–45 min in total.

### Pre-processing

Responses from the 52 participants (26 adults and 26 children) were pre-processed in order to extract reaction times and mark trials for accuracy. The point of highest intensity within the stimulus-initial inserted beep was identified using a customized script in *Praat* (Boersma and Weenink, 2022) and served as the anchor for calculating reaction times. The onset of each participant’s response to each sentence was marked with the help of Chronset (Roux et al., 2017), a tool for automated speech onset detection, and a customized script in *Praat* which monitored intensity changes. The automatic onset markings were examined and manually corrected by three trained phoneticians with partial cross-checking, while simultaneously marking accuracy of the responses, i.e., whether the spoken response was the same as the target word of the stimuli. Practice trials and three semantically ambiguous targets were removed post-hoc from the stimuli set. Further trials were discarded if the response recordings were of poor quality and/or when the precise speech onset could not be identified reliably (e.g., due to audio lag, background noise, failed recording of the beep, or overlap between the offset of the stimulus and response onset; 0.50%). Furthermore, trials were excluded if the participant repeated other words in the stimuli in addition to the target word (0.45%). This left a total of 5,946 out of 6,003 trials; Adults: 3,003 trials; Children: 2,943 trials. Responses that did not match the presented target word and replies of “I do not know” were marked as inaccurate responses and included in the accuracy analysis.

### Accuracy and Reaction Time Analyses

Regression analyses were conducted to analyze accuracy and reaction times using the statistical software R (version 2021.9.2.382; RStudio Team, 2021) and the lme4 package (version 1.1.27.1; Bates et al., 2015). Percent of inaccurate responses and mean reaction time by participant were not correlated (*r*(50) = 0.004, *p* = 0.488) and therefore Accuracy and Reaction Times were analyzed separately. In all models, categorical predictors were sum-to-zero coded and continuous predictors were centered. A mixed-effects logistic regression analysis was performed to model response accuracy. Reaction times of all accurate responses were analyzed with linear mixed-effects models after removing extreme values above 2,000 ms and below −400 ms (55 trials, 0.9%, *cf*. Mahler and Chenery, 2019) and subsequently removing outliers three standard deviations from the mean (70 outliers, 1.2%). Models were fitted first to the full dataset and then to adults and children separately. In addition, a separate model was fitted to test whether participants’ performance improved over the course of the experiment. Random effects (Subject and Item) and fixed effects were optimized with stepwise comparisons of model fit in the accuracy models, and with the “step” function in the reaction time models (lmerTest package [version 3.1.3], Kuznetsova et al., 2015). Significant *p*-values are reported at *p* < 0.05 (lmerTest package [version 3.1.3], Kuznetsova et al., 2015).

## Results

### Accuracy

A breakdown of participants’ mean percentage inaccuracy averaged by Condition (combined factors Acoustic Mask, Visual Mask, and Cloze Probability) is given in Supplementary Material 2. Inspection of inaccurate responses per Condition indicated that each combination of levels involving low Cloze Probability had a higher mean percentage inaccuracy than its equivalent combination with high Cloze Probability. In any given combination of levels, the children’s mean percentage of inaccurate responses was higher than the equivalent percentage for adults. Although accuracy overall was high, a substantial number of mistakes were made in low Cloze Probability sentences when both Acoustic Mask and Visual Mask were present, i.e., the least optimal combination of Conditions (Adults: 7.56%; Children: 9.49%). The largest percentage difference between adults and children was observed in the low Cloze Probability, Acoustic Mask only Condition (No Visual Mask), for which adults’ inaccurate responses (2.09%) were 5.89% lower than children’s (7.98%). Mean inaccuracies in all other Conditions not mentioned above were 4% or lower for both groups.

**Figure 3:**
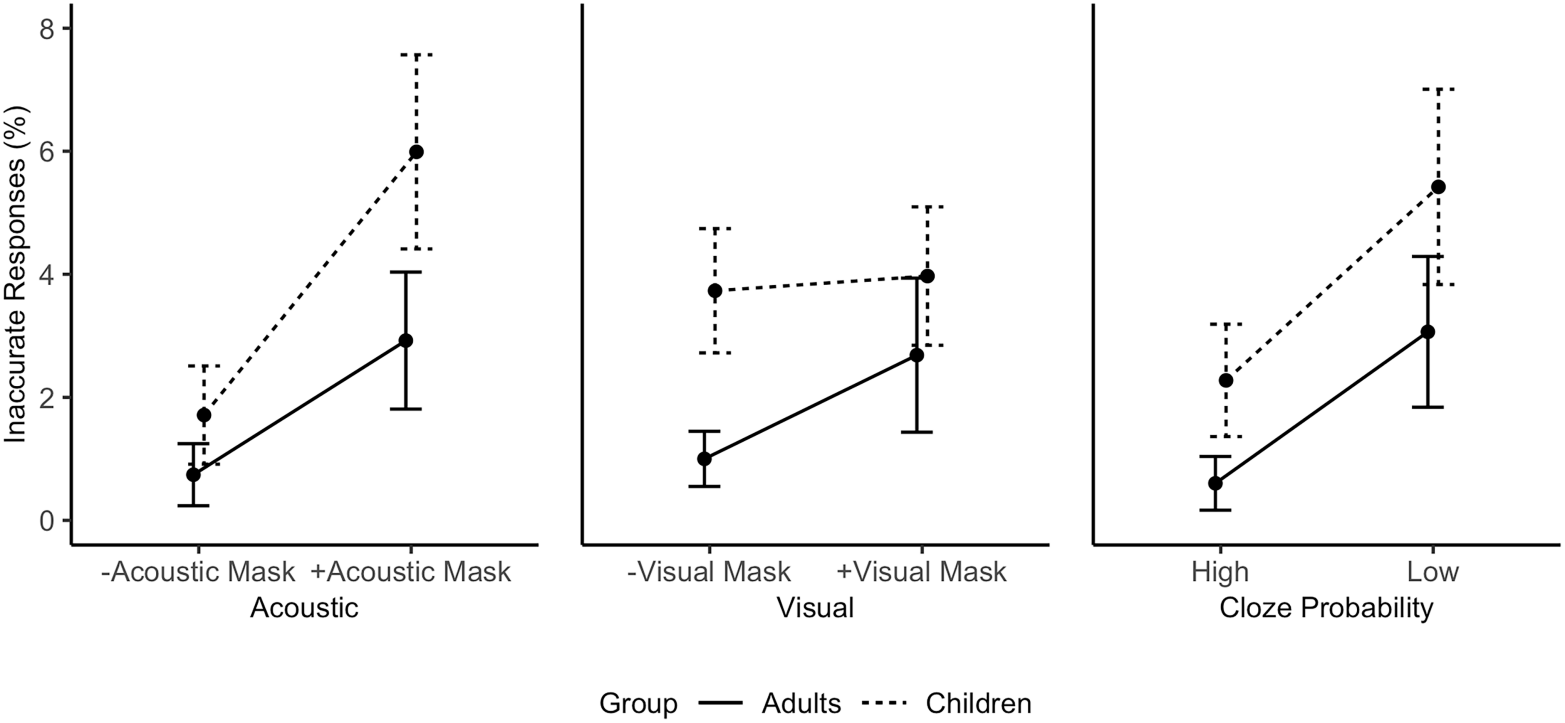
Individual main effects on subjects’ mean response accuracy in % averaged by Age Group based on the raw data (from left to right: Acoustic Mask Effect, Visual Mask Effect, Cloze Probability Effect).

The individual effects of Acoustic Mask, Visual Mask, and Cloze Probability were analyzed with mixed-effects logistic regression to model the binary outcome of accuracy. The raw accuracy data averaged by subjects are visualized in Figure 3 (% inaccurate responses). First, a maximal model was fitted to the full dataset. The fixed effects structure included Acoustic Mask (off/on), Visual Mask (off/on), Cloze Probability (high/low), Age Group (adults/children), and Trial Order (i.e., the order of stimulus presentation) as main effects, and six two-way interactions (Acoustic:Visual, Acoustic:Age Group, Visual:Age Group, Acoustic:Cloze Probability, Visual:Cloze Probability, and Age Group:Cloze Probability). Fixed effects were optimized through stepwise comparison of model fit. Random intercepts for Subject and Item, a by-Subject random slope for Cloze Probability, and a by-Item slope for the Visual Mask effect were retained in the full data accuracy model and optimized in the by-group models to avoid overfitting.

The optimized fixed model structure included the main effects Acoustic Mask, Visual Mask, Cloze Probability, and Age Group, as well as a two-way interaction between Visual Mask and Age Group (glmer(Accuracy ~ Acoustic Mask + Visual Mask + Cloze Probability + Age Group + Visual Mask*Age Group) + (1 + Cloze Probability|Subject) + (1 + Visual Mask|Item); Supplementary Material 3). On average, adults made fewer mistakes (1.82%) than children (3.86%) in the experiment (main effect Age Group, OR = 1.68, 95% CI [1.31, 2.14], *p* < 0.001). Inaccurate responses were more likely in sentences produced through an Acoustic Mask (OR = 2.06, 95% CI [1.68, 2.54], *p* < 0.001), sentences produced through a Visual Mask (OR = 1.38, 95% CI [1.12, 1.70], *p* = 0.002) and in low Probability sentences (OR = 1.85, 95% CI [1.40, 2.44], *p* < 0.001). The interaction between Visual Mask and Age Group (OR = 1.29, 95% CI [1.05, 1.57], *p* < 0.013) indicated that visual cues have a different effect on children’s and adults’ listening accuracy, motivating further analyses. Post-hoc pairwise comparisons (with Tukey adjustments) revealed that the adults were more likely to make inaccurate responses in the +Visual Mask condition than in the -Visual Mask condition (OR = 3.16, *z* = 3.27, *p* = 0.006). The same contrast was not significant for children (OR = 1.16, *z* = 0.66, *p* = 0.912). In addition, separate models for adults and children were fitted, starting with the same maximal fixed structure and random effects. The optimized adult and child models contained random intercepts for Subject and Item, as well as main effects of Acoustic Mask, Visual Mask, and Cloze Probability (no interactions; Table 2). The Acoustic Mask and low Cloze Probability led to significantly more inaccurate responses in both groups. The Visual Mask effect on response accuracy was significant in the adult group (OR = 1.69, 95% CI [1.20, 2.39], *p* = 0.003), but not in the child group (OR = 1.03, 95% CI [0.84, 1.27], *p* = 0.767).

**Table 2:** Optimized generalized mixed-effects regression models for adults and children, respectively, with accuracy as response variable.

Predictors	Adults			Children		
	OR	CI	*p*	OR	CI	*p*
Intercept	212.90	116.84–387.92	**<0.001**	55.64	39.99–77.41	**<0.001**
Acoustic mask	2.08	1.43–3.04	**<0.001**	2.08	1.61–2.67	**<0.001**
Visual mask	1.69	1.20–2.39	**0.003**	1.03	0.84–1.27	0.767
Cloze probability	2.36	1.51–3.71	**<0.001**	1.68	1.28–2.22	**<0.001**
Random effects						
*σ* ^2^	3.29			3.29		
*τ*_00 ITEM_	1.12			1.08		
*τ*_00 SUBJECT_	0.48			0.08		
ICC	0.33			0.26		
*N*_SUBJECT_	26			26		
*N*_ITEM_	234			234		
Observations	3,003			2,943		
Marginal *R*^2^/conditional *R*^2^	0.242/0.490			0.152/0.373		

Both models: glmer(accuracy ~ Acoustic Mask + Visual Mask + Cloze Probability) + (1|Subject) + (1|Item).p values <.05 are presented in bold.

### Reaction Times

A model was first fitted to the full dataset (*N* = 5,653) including random intercepts for Subject and Item, random slopes for Visual Mask effect and Cloze Probability by Subject, and random slopes for Visual Mask effect and Acoustic Mask effect by Item. Random effects, six interactions (Acoustic:Visual, Acoustic:Age Group, Visual:Age Group, Acoustic:Cloze Probability, Visual:Cloze Probability, and Age Group:Cloze Probability), and five main effects (Age Group, Cloze Probability, Visual Mask, Acoustic Mask, and Trial Order) were reduced with stepwise model comparison. The combined effects of audio-visual masking and Cloze Probability on mean reaction times are summarized in Supplementary Material 4.

The optimal full data model (lmer(Reaction Times ~ Acoustic Mask + Visual Mask + Cloze Probability + Age Group + Trial Order + Acoustic Mask*Visual Mask + Acoustic Mask*Cloze Probability + Cloze Probability*Age Group + (1 + Cloze Probability|Subject) + (1|Item)); Supplementary Material 5) with Subject and Item random intercepts and by-Subject random slope for Cloze Probability, included five main effects and three interactions (Marginal *R*^2^ = 0.05; Conditional *R*^2^ = 0.70). The model showed main effects of Acoustic Mask (*b* = −12.64, 95% CI [−18.18, −7.09], *p* < 0.001), Visual Mask (*b* = −13.11, 95% CI [−18.73, −7.48], *p* < 0.001), and Cloze Probability (*b* = −40.89, 95% CI [−53.88, −27.90], *p* < 0.001), respectively revealing significantly slower responses to speech produced through an Acoustic Mask compared to no Acoustic Mask (*M* = 511 ms, 95% CI [496, 525] vs. 491 ms, 95% CI [478, 505]), slower responses to Visually Masked than Visually Unmasked stimuli (*M* = 511 ms, 95% CI [497, 525] vs. 491 ms, 95% CI [477, 504]), and slower responses to targets with low Cloze Probability than high Cloze Probability (*M* = 541 ms, 95% CI [527, 555] vs. 462 ms, 95% CI [449, 476]). In addition, Trial Order, i.e., the order of stimulus presentation (*b* = −0.65, 95% CI [−0.81, −0.49], *p* < 0.001), and Age Group (*b* = −89.53, 95% CI [−168.41, −10.64], *p* = 0.026; Adults: *M* = 413 ms, 95% CI [402, 423]; Children: *M* = 594 ms, 95% CI [578, 610]) were significant main effects. The full dataset model further revealed significant interactions between Acoustic and Visual Mask effect (*b* = 6.98, 95% CI [1.34, 12.63], *p* = 0.015) and between Acoustic Mask and Cloze Probability (*b* = 6.57, 95% CI [1.03, 12.11], *p* = 0.020), as well as a marginal interaction between Age Group and Cloze Probability (*b* = −7.19, 95% CI [−14.46, −0.08], *p* = 0.052), motivating separate models for adults and children. The optimized models for adult data (*n* = 2,903) and child data (*n* = 2,750) are summarized in Table 3.

**Table 3:** Optimized linear mixed-effects models for adults and children, respectively, with reaction times as response.

Predictors	Adults			Children			*b*	CI	*p*	*b*	CI	*p*
Intercept	415.18	336.59–493.77	**<0.001**	593.95	456.42–731.47	**<0.001**
Acoustic mask	−10.65	−16.93 – −4.36	**0.001**	−13.99	−23.44 – −4.54	**0.004**
Visual mask	−10.31	−16.80 – −3.83	**0.002**	−13.35	−22.70 – −4.01	**0.005**
Cloze probability	−47.88	−59.67 – −36.09	**<0.001**	−32.84	−50.58 – −15.10	**<0.001**
Trial order	−0.65	−0.83 – −0.46	**<0.001**	−0.66	−0.93 – −0.39	**<0.001**
Acoustic*Visual	7.95	1.57–14.34	**0.015**			
Acoustic*Cloze probability	10.62	4.34–16.90	**0.001**			
Random effects						
*σ* ^2^	28866.64			59598.02		
*τ*_00_	6114.62_ITEM_			8225.59_ITEM_		
	40833.01_SUBJECT_			126388.25_SUBJECT_		
*τ*_11_				624.73_SUBJECT.PREDICT1_		
*ρ*_01_				0.17_SUBJECT_		
ICC	0.62			0.69		
*N*_SUBJECT_	26			26		
*N*_ITEM_	234			234		
Observations	2,903			2,750		
Marginal *R*^2^/conditional *R*^2^	0.040/0.635			0.010/0.697		

Adults’ model: lmer(Reaction Times ~ Acoustic Mask + Visual Mask + Cloze Probability + Trial Order + Acoustic Mask*Visual Mask + Acoustic Mask*Cloze Probability + (1|Subject) + (1|Item). Children’s model: lmer(Reaction Times ~ Acoustic Mask + Visual Mask + Cloze Probability + Trial Order + (1 + Cloze Probability|Subject) + (1|Item).p values < .05 are presented in bold.

**Figure 4:**
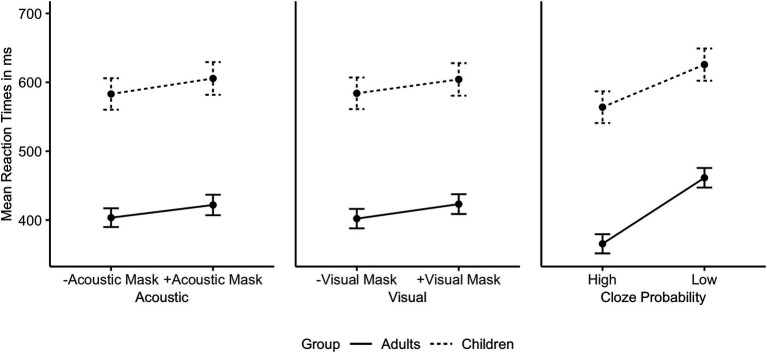
Individual main effects on mean reaction times in ms (with error bars) by Age Group based on the raw data (from left to right: Acoustic Mask Effect, Visual Mask Effect, Cloze Probability Effect).

**Figure 5:**
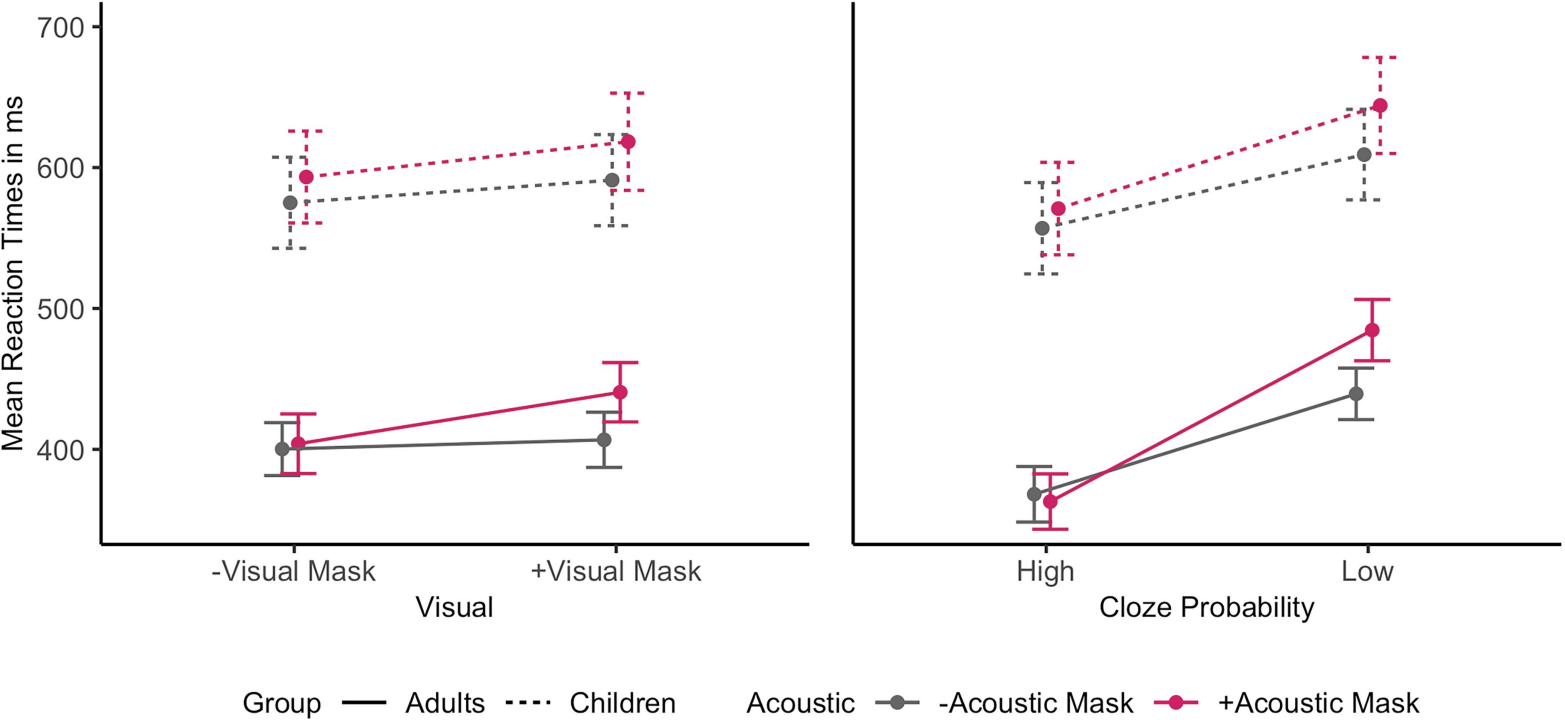
Interaction effects on mean reaction times in ms (with error bars) by Age Group based on the raw data (on the left: Acoustic Mask*Visual Mask; on the right: Acoustic Mask*Cloze Probability). Interactions in the mixed-model analysis were significant only for the adult comparisons (solid lines).

A by-Subject random slope for Cloze Probability improved the children’s model, but not the adults’ model. Figure 4 shows the individual main effects of Acoustic Mask, Visual Mask, and Cloze Probability by group. Both adults and children exhibited significant main effects of Acoustic Mask effect, Visual Mask effect, Cloze Probability, and Trial Order (*ps* < 0.01). The child model did not show any significant interactions. The adult model showed significant interactions between Acoustic Mask effect and Visual Mask effect (*b* = 7.95, 95% CI [1.57, 14.34], *p* = 0.015) as well as between Acoustic Mask effect and Cloze Probability (*b* = 10.62, 95% CI [4.34, 16.90], *p* = 0.001). Figure 5 shows the interactions between Acoustic Mask:Visual Mask and between Acoustic Mask:Cloze Probability by group (Figures 4, 5 are based on raw reaction time means in ms).

Post-hoc pairwise comparisons (with Tukey adjustment) of the Acoustic:Visual interaction revealed that the fully masked condition (i.e., +Acoustic Mask, +Visual Mask) was significantly slower than all other conditions (*ps* < 0.001). Post-hoc analyses performed on the Acoustic:Cloze Probability interaction revealed that an + Acoustic Mask had an effect on adults’ response latencies only in the low Cloze Probability context (*b* = 42.54, *t* = 4.62, *p* < 0.001), but not in the high Cloze Probability context (*b* = 0.05, *t* = 0.01, *p* = 1.00). Furthermore, the estimate sizes of these post-hoc comparisons indicated that high Cloze Probability reduced response latencies to a larger extent when listeners processed speech though the Acoustic Mask (+ Acoustic Mask; *b* = −117.00, *t* = −8.55, *p* < 0.001) than clear speech (–Acoustic Mask; *b* = −74.51, *t* = −5.49, *p* < 0.001).

**Figure 6:**
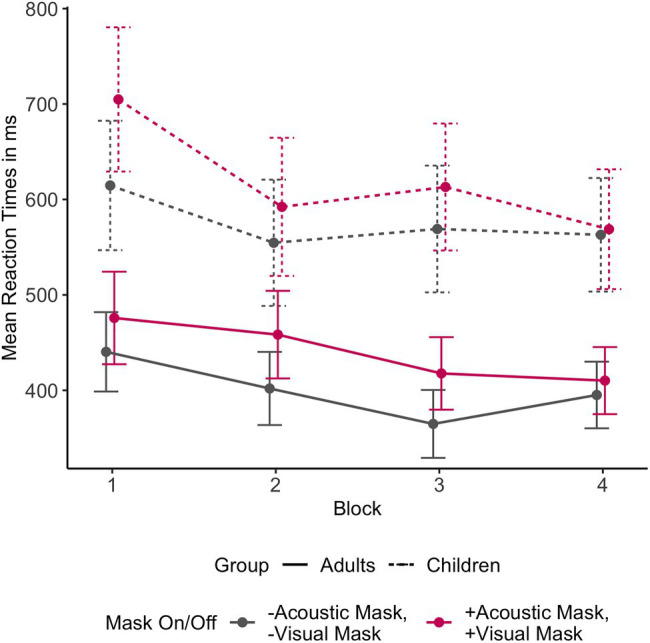
Mean reaction times across the four experiment blocks by Age Group based on the raw data, comparing fully masked + Acoustic Mask, + Visual Mask and fully unmasked – Acoustic Mask, – Visual Mask conditions.

A separate model tested whether participants improved their performance in perceiving masked speech over the course of the experiment. A random intercept only model was fitted with the predictors Trial Order, Mask Condition (both Acoustic and Visual Masks on vs. both off), and Age Group, and all respective two-way and three-way interactions between these effects. The best model fit showed significant main effects of Trial Order (*b* = −0.64, 95% CI [−0.88, −0.41], *p* < 0.001) and Mask Condition (*b* = −24.32, 95% CI [−32.53, −16.12], *p* < 0.001). No interactions were observed. Visual inspection of the data confirmed that participants responded faster to both Conditions — fully masked and fully unmasked — with practice (Figure 6).

In summary, the reaction time analysis showed that both adults and children gave significantly slower responses to target words presented through an acoustic face mask (audio manipulation), targets presented with the speaker wearing a visual face mask (video manipulation), and targets presented in low Cloze Probability sentences (semantic context manipulation). In addition, the adult-only model revealed significant interactions between Acoustic Mask and Visual Mask as well as between Acoustic Mask and Cloze Probability, indicating that adults utilized visual speech cues and semantic predictions to fully compensate for acoustic face mask effects.

## Discussion

### Summary of Findings

The present study used an internet-based cued shadowing paradigm to examine how audio-visual language is processed through face masks, measuring both response accuracy and response latencies. The aim of the study was to unravel the effects of acoustic masking, visual masking, and semantic context on 8–12-year-old children’s and adults’ speech processing. With a fully crossed 2 × 2 × 2 design, the relative contributions of acoustic changes and removal of visual cues through face masks were dissociated and further modulated by varying semantic predictability of sentence-embedded target words.

Contrary to the prediction that face mask speech will only affect response latencies (P1), in the present study the response accuracy of both adults and children was also reduced under masked conditions. For adults, this adverse mask effect on language comprehension stemmed from both the acoustic degradation and the visual obstruction, whereas children’s accuracy was only affected by the Acoustic Mask. This was indicated by a significant interaction between Visual Mask and Age Group in the full data model, as well as the presence of a Visual Mask effect in the adults’ model, but not in the children’s model. In other words, children were about equally likely to make a mistake with and without the presence of the speaker’s visual face mask, whereas adults were less likely to make mistakes in the presence of visual cues. This finding suggests that removal of visual cues is less detrimental to children’s language comprehension than has previously been assumed (UK Government, Department for Education, 2022). However, it is important to note that even in the worst listening condition (acoustic mask, visual mask, and low predictability) inaccuracy was below 10%. High semantic predictability significantly improved adults’ and children’s response accuracy under all conditions. Overall, the impact of face masks on speech intelligibility in the absence of noise seems mild.

With respect to response latencies, all three main factors of interest, i.e., Acoustic Mask, Visual Mask, and Cloze Probability, significantly modulated children’s and adults’ response times (P2). Focusing on responses in the low Cloze Probability context, i.e., the condition with minimal top-down influence, speech produced through an acoustic face mask led to slower responses compared to no mask (34 ms for adults, 29 ms for children), as did a visual face mask (21 ms for adults, 8 ms for children) compared to no mask. Averaging across all audio-visual conditions, a comparatively strong effect of semantic predictability was attested in the faster responses in high Cloze Probability contexts (95 ms for adults, 62 ms for children).

Furthermore, three significant interactions in the reaction time data revealed differences between children and adults (P3): An interaction between Acoustic Mask and Visual Mask and subsequent post-hoc comparisons revealed that only combined acoustic and visual face masks significantly affected adults’ reaction times, on average leading to 78 ms slower responses than to clear speech in low Cloze Probability contexts. When adults were presented with only an Acoustic Mask or only a Visual Mask, they were able to compensate fully for the effect with the other modality. This interaction was not present in the child data.

Children and adults also differed in the way they integrated the audio-visual signal with top-down semantic information. A significant interaction between Cloze Probability and Age Group indicated that adults on the whole were more efficient in using semantic context for predictive speech processing, reflected in larger reaction time differences between low and high Cloze Probability sentences. Adults’ slightly more efficient use of semantic information was also reflected in a significant interaction between Cloze Probability and Acoustic Mask in the adults’ model, resulting in the absence of an Acoustic Mask effect in sentences with high Cloze Probability in this group. This interaction was not present in the children’s model. Visual inspection of the interaction graphs (Figure 5) suggests that children are not yet as proficient in this semantic compensation mechanism. This interpretation is supported by the fact that semantic predictability contributed to the children’s model as a random slope effect, suggesting that some children may be more advanced in their use of semantic cues for degraded speech compensation than others.

Accuracy and reaction time results taken together suggest that both adults and children find processing face mask speech more cognitively demanding than processing clear speech. However, prior to the perception experiment, adult participants judged mask speech processing as more tiring and more difficult than parents did for their children (*cf*. 2.1 Participants). From the perspective of an outside observer, children’s speech comprehension difficulties most likely only become apparent when misunderstandings occur. Given the limited impact of face masks on comprehension accuracy relative to processing speed, parents might therefore not be fully aware of the increased cognitive demand masks pose for children.

Separate analysis of the reaction times across the course of the experiment (Trial Order) suggests that all participants improved (i.e., became faster at responding) with practice, and this effect was true regardless of masking condition. A significant interaction between Mask Condition and Trial Order was not found in the present study. However, previous research suggests that listeners are able to adapt to suboptimal listening conditions, such as background noise (Cervera and Gonzalez-Alvarez, 2007; Marrufo-Pérez et al., 2020). More research is needed to understand whether listeners adapt to face mask speech over time in a similar fashion, leading to a reduced effect of face masks on processing speed, which is an important factor in assessing the severity of face mask effects on speech comprehension.

### Integration of Acoustic, Visual, and Semantic Cues

Overall, the results show that face mask speech affected both children’s and adults’ speech processing, with similar effect sizes for Visual and Acoustic Masks on response latencies. With respect to the acoustic cues, the results of the present study suggest that acoustic degradation of face mask speech resembles other types of acoustically degraded speech which increase cognitive demands for operations such as language processing and thus lead to slower responses and a higher probability of making word identification mistakes (Peelle, 2018). However, the findings contradict previous suggestions that effects of face masks on intelligibility may be predominantly driven by the removal of visual cues provided by the speaker’s lips (e.g., Llamas et al., 2008). The relatively small visual gain observed in quiet conditions for adults in the current study may be due to the presentation of speaker videos in all conditions. Most studies testing audio-visual gain compare an audio-visual condition (i.e., audio and video are displayed together) to an audio-only condition (i.e., no video is displayed; Schwartz et al., 2004; Wightman et al., 2006; Ross et al., 2007, 2011; Llamas et al., 2008; Barutchu et al., 2010; Fraser et al., 2010; Maidment et al., 2015). In the current study, all conditions featured a video of the speaker, with and without face mask. Consequently, some visual cues were still available to the listeners even under the Visual Mask condition, e.g., small movements of the face mask, eyebrow movements, and important social cues from the eyes (*cf*. Emery, 2000; Brooks and Meltzoff, 2002). This seems to suggest that a real-time speaker’s image accompanying the speech, not necessarily including clear articulator movements, is beneficial to multisensory speech perception. Furthermore, visual cues may become more important under noisier conditions (Ross et al., 2007; Ma et al., 2009; Brown et al., 2021).

In the current study, the integration of auditory and visual information differed between children and adults. Adults were able to compensate for the signal loss in one modality (visual or acoustic) by relying more on the other, resulting in similar processing times to the condition when both modalities were intact. Only when Acoustic Mask and Visual Mask were both present (as is the case for most face coverings) did face mask speech significantly increase processing time for adults. The audio-visual interaction also suggests that adults benefitted more greatly from visual articulatory cues when the acoustic signal was masked. This is in line with previous literature on audio-visual integration. Although adults tend to pay more attention to the eyes than the mouth during speech processing (Lansing and McConkie, 2003), they can shift their attention more toward lip-reading to compensate for cognitively demanding listening conditions, for example when listening to speech in noise (Vatikiotis-Bateson et al., 1998), processing second languages or unfamiliar languages (Barenholtz et al., 2016; Birulés et al., 2020), or attending to different speakers in quick succession (Buchan et al., 2008). These studies demonstrate the “inverse effectiveness” principle in multisensory perception (Stein and Meredith, 1993), which predicts that audio-visual enhancement increases for poorly perceptible unisensory signals (Holmes, 2007; van de Rijt et al., 2019). In contrast to adults, no interaction between Acoustic and Visual Masks was found in the children’s data, and instead, each modality had an independent effect on children’s processing times. The present findings align with previous research suggesting that children up to 16 years of age are less proficient in using visual information in communication than adults (Massaro et al., 1986; Wightman et al., 2006), especially when the speech signal is degraded or presented in noise (Barutchu et al., 2010; Ross et al., 2011). However, it has been shown that toddlers are already attentive to visual cues from the mouth to assist their language acquisition (Morin-Lessard et al., 2019), and allocate even more attention to the mouth in adverse listening conditions (Król, 2018). This suggests that children are indeed interested in visual linguistic cues, but have not fully acquired the same compensatory mechanism as adults whereby a moderate signal loss in one modality can be largely compensated by the other. More exposure to audio-visual stimuli might be necessary to achieve adult-like multi-modal integration.

The manipulation of Cloze Probability as an approximation of predictive semantic processing, and its interaction with visual and acoustic face mask effects, provides information on the integration of top-down and bottom-up processing of face mask speech for the first time. Cloze Probability had a larger effect on accuracy and reaction times than Acoustic and Visual Mask effects, across both children and adults, allowing listeners in the present study to process semantic cues effectively even under adverse audio-visual masking conditions. These results are consistent with evidence suggesting that higher-level, top-down processing is unaffected by mildly degraded bottom-up input (Davis and Johnsrude, 2003, 2007; Peelle et al., 2010; McGettigan et al., 2014; Sohoglu and Davis, 2016; Peelle, 2018). In fact, adult listeners not only had access to semantic processing mechanisms, but relied more strongly on these predictive processes under masked conditions, as indicated by an interaction between Acoustic Mask and Cloze Probability in the adult reaction time model. Enhanced pre-activation of stored semantic knowledge while processing the imperfect, masked speech signal led to the absence of face mask effects in high Cloze Probability contexts for adult listeners. In sum, the present study provides evidence that predictive semantic processing aids accurate and efficient comprehension of face mask speech as a compensatory mechanism.

Although children in this study were less efficient in this type of semantic-predictive compensation (in line with Mahler and Chenery, 2019), they were still able to use semantic information to their benefit, as reflected by a main effect of Cloze Probability. This finding aligns with previous research suggesting that children as young as two years of age make use of semantic cues, though less efficiently than adults (Friedrich and Friederici, 2004). While children had equal access to semantic prediction mechanisms across all conditions in the same way as adults, the absence of a significant interaction between Acoustic Mask and Cloze Probability in the child reaction time model suggests that, in contrast to adults, children do not activate semantic knowledge more strongly under masked conditions. However, this difference could be partly due to variability within the children’s group, as indicated by the significant Cloze Probability random slope in the children’s reaction time model. Visual inspection of the raw data revealed a reduced difference between Acoustic Mask and unmasked condition in high Cloze Probability contexts (Supplementary Material 4), supporting the interpretation that developmental differences led to a smaller, nonsignificant interaction effect, rather than a complete absence of semantic compensation. Therefore, the results are most in line with language processing accounts that attribute integration differences of linguistic cues to a lack of language experience, rather than complete absence of adult-like processing mechanisms (Mahler and Chenery, 2019).

### Practical Implications in Classroom Settings

The results have a number of practical implications for communicating with face masks in classroom environments. The present study indicates that face mask speech leads to more mishearings and slower responses by both children and adults, but predominantly so in low predictability contexts. Crucially, this means that adequate semantic context could help to minimize any adverse mask effects. The reduced impact of masks on intelligibility and processing speed when adequate semantic context is given suggests that face masks do not strongly impede 20–60-year-old adults’ and 8–12-year-old children’s understanding of speech when listening to a single adult speaker (e.g., a teacher) wearing a mask. However, given the indications we found that masks increase cognitive demand for speech processing, and as a result increase listening effort, pupils may find it helpful to be given more context, regular breaks, and more time to process new content and respond to questions. Furthermore, explicit awareness of the importance of contextual information can be used by teachers to their advantage by capitalizing on the kinds of creative best practices they already use to aid pupils’ understanding. In particular, this may include building upon students’ prior knowledge and informing children (and parents, where relevant) of the upcoming lesson topics, providing as much (semantic) context as possible, especially for new topics (i.e., avoiding contextually uninformative sentences similar to those used in the low predictability condition in this study), checking students’ understanding of the content more frequently, and using visual aids (text, images, and written key words).

Interestingly, visual face mask effects in the present study were comparatively small, possibly so because visual cues from the upper half of the speaker’s face were present in all conditions. This suggests that being able to see the (mask-wearing) teacher’s facial expressions from the noncovered part of the face may be advantageous. While more research is necessary to reach any firm conclusions, it appears that transparent masks may not be the best solution for teaching hearing children: First, the present findings suggest that children do not achieve the compensating effects from observing lip movements to the same degree as adults. Furthermore, although children pay attention to lip movements to obtain additional linguistic cues when the sound is degraded (Król, 2018), transparent masks have a tendency to become obscured by condensation (Brown et al., 2021) and exhibit worse acoustic performance than other mask types (Corey et al., 2020). In addition, previous studies that have compared masked speech intelligibility in quiet and noisy conditions (Brown et al., 2021) indicate that reduction of background noise is likely to be advantageous for listening comprehension when face masks are worn (e.g., by avoiding parallel conversations in groups).

## Conclusion

The present study indicates that the difficulties people sometimes experience when listening to speech produced through a cloth face mask are likely to stem from both the acoustic degradation of the speech signal and the removal of visual information of the lower half of the face. However, adults only responded significantly more slowly when both the acoustic and the visual signal were degraded. Children and adults made use of acoustic speech cues to a similar degree, but children were less proficient than adults in using visual speech information. Provision of adequate contextual information through semantic cues on the sentence-level reduced audio-visual mask effects for both children and adults. As a result, adults were largely able to compensate for the acoustically degraded mask speech in high predictability contexts. Children processed semantic cues in a similar fashion to adults, but they were less efficient in using them as a compensatory strategy for the degraded speech signal. Since the current study focused on quiet listening conditions and native speakers of English without any hearing, seeing, or language-related difficulties, more work is needed for other listening conditions and populations. Due to the simplicity of the task, internet-based cued shadowing has great potential for the inclusion of diverse populations in research, e.g., children with varying levels of hearing or learning difficulties, minority groups, and hard-to-reach communities.

## Data Availability Statement

The datasets presented in this study can be found in online repositories. The names of the repository/repositories and accession number(s) can be found at: Open Science Framework (OSF), “PerMaSC: Speech Perception through Masks in School Contexts:” https://doi.org/10.17605/OSF.IO/ETVDG.

## Ethics Statement

The studies involving human participants were reviewed and approved by University of Cambridge Faculty of Modern and Medieval Languages and Linguistics Research Ethics Committee. Written informed consent to participate in this study was provided by the participants’ legal guardian/next of kin. Written informed consent was obtained from the individual(s) for the publication of any potentially identifiable images or data included in this article.

## Author Contributions

JSc and KL conceived and designed the study. KL and JSi prepared the acoustic-visual stimuli and wrote sections of the manuscript. JSc implemented the online experiment and wrote the first draft of the manuscript. KL, JSi, JSc and KM recruited the participants. KL developed the pipeline and tools for data processing. KL, JSc, JSi and YZ processed the data. JSc, JSi performed the statistical analysis. JSc, KL, JSi, BP, KM, and EB-W interpreted the data together. KM, EB-W, BP, YZ, and JG contributed to the development of the study and manuscript revision. All authors contributed to the article and approved the submitted version.

## Funding

This research was funded by a grant for a project entitled Speech Perception through Masks in School Contexts (PerMaSC) from the Cambridge Language Sciences Incubator Fund (Principal Investigator: KM; Lead Applicants: JSc and KL) and a UKRI grant to JSc [ES/J500033/1].

## Conflict of Interest

The authors declare that the research was conducted in the absence of any commercial or financial relationships that could be construed as a potential conflict of interest.

## Publisher’s Note

All claims expressed in this article are solely those of the authors and do not necessarily represent those of their affiliated organizations, or those of the publisher, the editors and the reviewers. Any product that may be evaluated in this article, or claim that may be made by its manufacturer, is not guaranteed or endorsed by the publisher.
