# Supplementary material for: Semantic Cues Modulate Children’s and Adults’ Processing of Audio-Visual Face Mask Speech
Source: Front Psychol. 2022 Jul 19;13:879156. doi: 10.3389/fpsyg.2022.879156 (PMC9343587; doi:10.3389/fpsyg.2022.879156)
Supplement: Supplementary file 1 [file Data_Sheet_1.PDF]

Supplementary Material 1

List of Stimuli

High Cloze Probability	Low Cloze Probability
The cow was milked in the barn.	Tom could not discuss the barn.
The boat sailed across the bay.	Mr. Smith knew about the bay.
The chicken pecked corn with its beak.	She's glad Bill called about this beak.
The torch casts a bright beam.	She's discussing the right beam.
Follow this road around the bend.	I'm glad you heard about the bend.
The guilty one should take the blame.	David should consider the blame.
The bomb exploded with a blast.	The class will consider a blast.
The soup was served in a bowl.	You're glad she called about a bowl.
Spread some butter on the bread.	He could discuss the bread.
The girl swept the floor with the broom.	Ruth's Grandmother discussed the broom.
Tom fell down and got a bruise.	Sue was interested in a bruise.
For your birthday I baked this cake.	Tom wants to know about this cake.
The cow gave birth to the calf.	She hopes Jane called about the calf.
Peter dropped in for a chat.	Bill had a problem with a chat.
Cut the meat into small chunks.	I did not know about small chunks.
Ann works in the bank as a clerk	Bob was considering a clerk.
The car drove off the cliff.	They had a problem with the cliff.
We heard the ticking of the clock.	Tom is considering the clock.
The detectives searched for a clue.	The man spoke about a clue.
The team was trained by the coach.	Bill heard Tom called about the coach.
The boat sailed along the coast.	Miss Brown might consider the coast.
Let's decide by tossing a coin.	Jane has a problem with a coin.
Paul was arrested by the cops.	Ruth hopes Bill called about the cops.
No one was injured in the crash.	I want to speak about the crash.
The ship's captain summoned the crew.	She wants to talk about the crew.
The baby slept in his crib.	He can't consider his crib.
The farmer harvested his crop.	I want to know about his crop.
The king wore the crown.	I've been considering the crown.
The car was parked at the curb.	Betty doesn't discuss the curb.
He was hit by a poisoned dart.	He hasn't considered that dart.
The sailor swabbed the deck.	He hears she asked about the deck.
They tracked the lion to his den.	Bill cannot consider his den.

How did your car get the dent?	David has discussed the dent.
The workers are digging a ditch.	You want to talk about a ditch.
The aeroplane went into a dive.	The old man discussed a dive.
The little girl cuddled the doll.	Miss Black knew about the doll.
The bird of peace is the dove.	Peter could consider the dove.
The doctor prescribed the drug.	She has known about the drug.
They marched to the beat of the drum.	We hear she called about the drum.
The candle burned with a bright flame.	The girl should consider the right flame.
The shepherds guarded their flock.	They should have discussed their flock.
The pond was full of frogs.	The woman talked about frogs.
It was stuck together with glue.	Tom has not dealt with glue.
The hockey player scored a goal.	She has a problem with a goal.
The bride wore a white gown.	The girl should not discuss that gown.
She faced them with a foolish grin.	Miss Brown will speak about his grin.
The watchdog gave a growl.	I had thought about a growl.
The farmer baled the hay.	Tom discussed the hay.
Instead of a fence plant a hedge.	He was interested in a hedge.
The chicks followed the mother hen.	Bill didn't discuss a hen.
The rancher rounded up his herd.	Ruth will consider this herd.
I can't guess so give me a hint.	We have not thought about a hint.
Bob stood with his hands on his hips.	Bob hopes he heard about his hips.
The honey bees swarmed round the hive.	They might have considered the hive.
The guests were welcomed by the host.	Bill heard we asked about the host.
I gave her a kiss and a hug.	David does not discuss a hug.
The cookies were kept in the jar.	He's glad you called about the jar.
At breakfast he drank the juice.	We should have considered the juice.
Throw out all this useless junk.	Ruth hopes she called about this junk.
The boy gave the football a kick.	The old man considered a kick.
I cut my finger with the knife.	I am thinking about the knife.
Unlock the door and turn the knob.*	We spoke about the knob.*
She held the baby on her lap.	Miss Black thought about her lap.
To open the jar, twist the lid.	The woman knew about the lid.
This key won't fit in the lock.	We hear you called about the lock.
The cabin was made of logs.	Harry had thought of logs.
The cigarette smoke filled his lungs.	The old man talked about his lungs.
We're lost so let's look at the map.	I should have considered the map.
Please wipe your feet on the mat.	Peter has considered the mat.
She cooked him a hearty meal.*	Ruth is speaking about the charity meal.*
Wipe the floor with the mop.	He doesn't discuss the mop.

The cats love to chase the mouse.	The man could not discuss the mouse.
Tim was drinking hot chocolate from a mug.	Peter should speak about a mug.
He caught the fish in a net.*	Paul should know about a net.*
Tighten the belt by a notch.	The woman considered a notch.
Tear off some paper from the pad.	Mr. Black knew about the pad.
My son has a dog for a pet.	They heard I called about a pet.
For dessert he baked a pie.	Ruth must have known about a pie.
The sand was heaped in a pile.	I've spoken about a pile.
The sick child swallowed the pill.	Tom had spoken about the pill.
This furniture was made of pine.	She couldn't think of pine.
The story had a clever plot.	You're discussing a plot.
Raise the flag up the pole.	Bob could consider the pole.
The best entry will win the prize.	We are speaking about the prize.
The shipwrecked sailors built a raft.	Peter knows about a raft.
The landlord raised the rent.	They hope he heard about the rent.
The cup had a chip on the rim.	Paul could not consider the rim.
He tossed the drowning man a rope.	Miss Black could have discussed a rope.
The children were all lined up in a row. (as in "no")	Miss Smith couldn't discuss a row. (as in "no")
On the beach we play in the sand.	Miss Brown shouldn't discuss the sand.
The cut on his knee formed a scab.	The boy would discuss the scab.
Watermelons have lots of seeds.	You have not thought of seeds.
To store his wood he built a shed.	We hear they asked about a shed.
The shepherd watched his flock of sheep.	They often think of sheep.
The scarf was made of silk.	Tom won't think of silk.
She shortened the hem of the skirt.	Nancy didn't discuss the skirt.
The sport shirt has shorter sleeves.	Nancy had considered the sleeves.
Get the bread and cut me a slice.	Jane did not speak about a slice.
Keep your broken arm in a sling.	Ruth wants to speak about a sling.
Paul hit the water with a splash.	Bob has discussed a splash.
He wiped the sink with the sponge.	I haven't discussed the sponge.
The thread was wound on a spool.	The man could consider a spool.
Stir your coffee with the spoon.	Bob could have known about the spoon.
Football is a dangerous sport.	Tom could have thought about this sport.
Kill the bugs with this spray.	Mary had considered this spray.
The sandal has a broken strap.	They were interested in one strap.
Crocodiles live in muddy swamps.	The girl knows about many swamps.
The duck swam with the white swan.	Tom will discuss that swan.
Ruth poured herself a cup of tea.	Miss White often thinks of tea.
We camped out in the tent.	Bob has considered the tent.

The house was robbed by the thief.	The old woman discussed the thief.
A rose bush has prickly thorns.	The boy can't talk about many thorns.
I've got a cold and a sore throat.	He is considering a throat.
Metal cans were made with tin.	He had a problem with tin.
The dog followed the trail.	Harry will consider the trail.
The mouse was caught in the trap.	The boy might consider the trap.
The furniture was delivered by a truck.	We've spoken about a truck.
Paul took a bath in the tub.	Miss Smith knows about the tub.
Household goods are moved in a van.	You heard Jane called about a van.
The candle flame melted the wax.	Paul can't discuss the wax.

* Stimuli removed from the analysis due to ambiguity.

Supplementary Material 2

Summary of participants' mean percentage response inaccuracy by combined Audio-Visual Conditions (+Mask / –Mask), Cloze Probability, and Age Groups.

Masking Conditions	Cloze Probability	Participants' mean inaccuracy in per Condition in % (standard deviation)			
		**Adults**	*****	**Children**	*****
**+Acoustic Mask +Visual Mask**	**High**	1.39 (3.50)	+1.13	2.50 (4.53)	+1.18
	**Low**	7.56 (8.41)	+6.45	9.49 (10.46)	+7.77
**+Acoustic Mask –Visual Mask**	**High**	0.51 (1.81)	+0.25	4.00 (4.46)	+2.68
	**Low**	2.09 (3.20)	+0.98	7.98 (7.95)	+6.26
**–Acoustic Mask +Visual Mask**	**High**	0.26 (1.31)	+0	1.21 (2.94)	-0.11
	**Low**	1.38 (2.91)	+0.27	2.68 (4.44)	+0.96
**–Acoustic Mask –Visual Mask**	**High**	0.26 (1.31)		1.32 (3.34)	
	**Low**	1.11 (2.66)		1.72 (3.23)	

Note: * Mean inaccuracy differences compared to the respective Cloze Probability condition of the **-Acoustic Mask, –Visual Mask** condition.

Supplementary Material 3

Generalised linear mixed-effects regression fitted to the full dataset with accuracy as response variable.

**Predictors**	**Odds Ratios**	**CI**	***p***
Intercept	107.65	77.94 – 148.69	**< 0.001**
Acoustic Mask	2.06	1.68 – 2.54	**< 0.001**
Visual Mask	1.38	1.12 – 1.70	**0.002**
Cloze Probability	1.85	1.40 – 2.44	**< 0.001**
Age Group	1.68	1.31 – 2.14	**< 0.001**
Visual Mask * Age Group	1.29	1.05 – 1.57	**0.013**
**Random Effects**			
σ^2	3.29		
τ_{00} ITEM	1.01		
τ_{00} SUBJECT	0.19		
τ_{11} ITEM.VIDEO1	0.08		
τ_{11} SUBJECT.PREDICT1	0.18		
ρ_{01} ITEM	0.21		
ρ_{01} SUBJECT	-0.08		
ICC	0.31		
N SUBJECT	52		
N ITEM	234		
Observations	5946		
Marginal R^2 / Conditional R^2	0.220 / 0.458		

Model: glmer(Accuracy ~ Acoustic Mask + Visual Mask + Cloze Probability + Age Group + Visual Mask*Age Group) + (1 + Cloze Probability|Subject) + (1 + Visual Mask|Item).

Supplementary Material 4

Summary of mean reaction times in ms with 95% confidence intervals by combined Audio-Visual Conditions (+Mask / –Mask), Cloze Probability, and Age Groups.

Masking Conditions	Cloze Probability	Mean Reaction Times per Condition in ms			
		**Adults**	*	**Children**	*
**+Acoustic Mask +Visual Mask**	**High**	378, CI=[350,406]	+6	586, CI=[537,634]	+41
	**Low**	507, CI=[477,538]	+78	655, CI=[606,704]	+50
**+Acoustic Mask –Visual Mask**	**High**	348, CI=[320,376]	-24	556, CI=[512,601]	+11
	**Low**	463, CI=[432,494]	+34	634, CI=[586,681]	+29
**–Acoustic Mask +Visual Mask**	**High**	364, CI=[335,392]	-8	569, CI=[523,615]	+24
	**Low**	450, CI=[423,476]	+21	613, CI=[568,658]	+8
**–Acoustic Mask –Visual Mask**	**High**	372, CI=[345,400]		545, CI=[499,590]	
	**Low**	429, CI=[403,454]		605, CI=[560,651]	

Note: * Mean reaction time differences compared to the respective Cloze Probability condition of the **-Acoustic Mask, –Visual Mask** condition.

Supplementary Material 5

Linear mixed-effects regression fitted to the full dataset with reaction times as response variable.

**Predictors**	***b***	**CI**	***p***
Intercept	505.01	425.39 – 584.62	**< 0.001**
Acoustic Mask	-12.64	-18.18 – -7.09	**< 0.001**
Visual Mask	-13.11	-18.73 – -7.48	**< 0.001**
Cloze Probability	-40.89	-53.88 – -27.90	**< 0.001**
Trial Order	-0.65	-0.81 – -0.49	**< 0.001**
Age Group	-89.53	-168.41 – -10.64	**0.026**
Acoustic * Visual Mask	6.98	1.34 – 12.63	**0.015**
Acoustic * Cloze Probability	6.57	1.03 – 12.11	**0.020**
Cloze Probability * Age Group	-7.19	-14.46 – 0.08	0.052
**Random Effects**			
σ^2	43978.62		
$\tau_{00\ \text{ITEM}}$	7039.89		
$\tau_{00\ \text{SUBJECT}}$	83786.58		
$\tau_{11\ \text{SUBJECT.PREDICT1}}$	304.23		
$\rho_{01\ \text{SUBJECT}}$	0.22		
ICC	0.67		
N_{SUBJECT}	52		
N_{ITEM}	234		
Observations	5653		
Marginal R^2 / Conditional R^2	0.073 / 0.698		

Model: lmer(Reaction Times ~ Acoustic Mask + Visual Mask + Cloze Probability + Age Group + Trial Order + Acoustic Mask*Visual Mask + Acoustic Mask*Cloze Probability + Cloze Probability*Age Group + (1 + Cloze Probability|Subject) + (1|Item).
